# Supplementary material for: Adipocyte HSL is required for maintaining circulating vitamin A and RBP4 levels during fasting
Source: EMBO Rep. 2024 May 20;25(7):8. doi: 10.1038/s44319-024-00158-x (PMC11239848; doi:10.1038/s44319-024-00158-x)
Supplement: Supplementary file 1 — Appendix [file 44319_2024_158_MOESM1_ESM.pdf]

Appendix

table of content

Appendix Figure S1. Detection of TTR monomers in boiled serum and liver protein samples. p2
Appendix Figure S2. Effect of fasting on mouse organ/tissue weights.. p3
Appendix Figure S3. Effect of global and adipocyte-specific loss of HSL on plasma RBP4 levels in *ad libitum*-fed mice. p4
Appendix Table S1. siRNA oligonucleotides sequences (5'-3') p5
Appendix Table S2. qPCR primer oligonucleotide sequences (5'-3') p5
Appendix Table S3. Antibodies p5

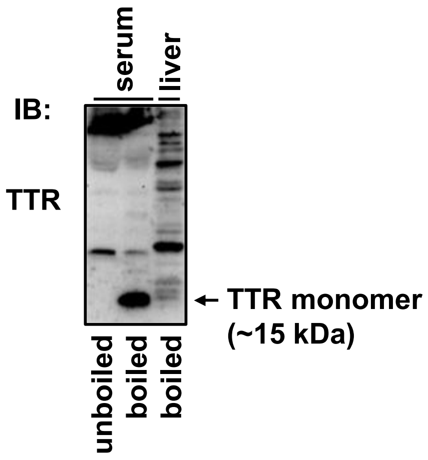

Appendix Figure S1. Detection of TTR monomers in boiled serum and liver protein samples. TTR abundance in serum and liver was analyzed by immunoblotting after boiling samples for 20 min prior loading. Unboiled serum served as negative control.

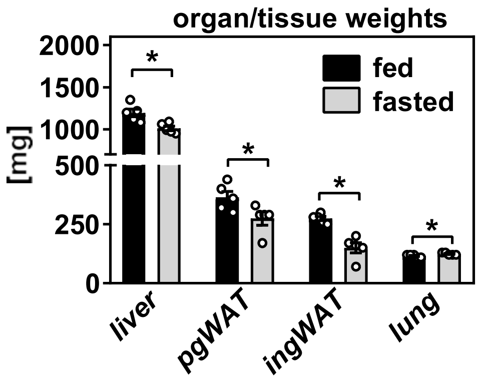

Appendix Figure S2. Effect of fasting on mouse organ/tissue weights. Organ weights of mice fed *ad libitum* or fasted for 24 hours were determined. Data information: Data are represented as individual data points of n=5,5 biological replicates and mean ± sem and *P<0.05 vs. *ad libitum*-fed mice using an unpaired two-tailed Student's t test.

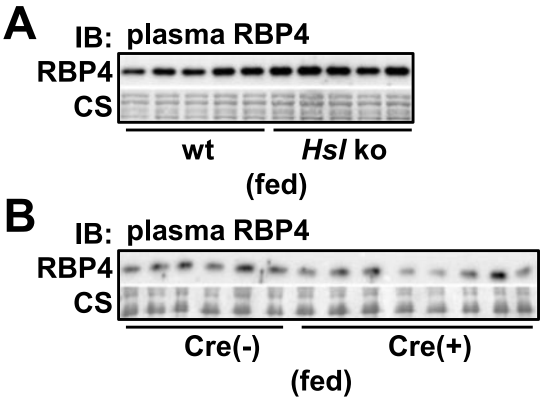

Appendix Figure S3. Effect of global and adipocyte-specific loss of HSL on plasma RBP4 levels in *ad libitum*-fed mice. Plasma RBP4 levels in *ad libitum*-fed (A) wt and *Hsl* ko mice and (B) *ADIPOQ*-Cre(-) and Cre(+) mice with floxed *Hsl* alleles was determined by immunoblotting. Coomassie staining (CS) served as loading control.

Appendix Tables

Appendix Table S1. siRNA oligonucleotides sequences (5'-3')

target	sense	antisense
siControl (non-targeting)	UAGCGACUAAACACAUCAA(UU)	UUGAUGUGUUUAGUCGCUA(UU)
siFoxo1_1	GCACCGACUUUAUGAGCAA(UU)	UUGCUCAUAAAGUCGGUGC(UU)
siFoxo1_2	GGACAACAACAGUAAAUUU(UU)	AAAUUUACUGUUGUUGUCC(UU)

Appendix Table S2. qPCR primer oligonucleotide sequences (5'-3')

gene/region	usage	forward	Reverse
mCycloB	qPCR	GGCTCCGTCGTCTTCCTTTT	ACTCGTCCTACAGATTCATCTCC
mCyp26a1	qPCR	TCTCCAACCTGCACGATTCC	CGGCTGAAGGCCTGCAT
mCyp26b1	qPCR	TCATCGGAGAGACTGGTCACT	GGTGCTCACTAGCTGGTGTTC
mIgfbp1	qPCR	ACGAGCACCTTGTTCAGCTC	GCAGCTGCTCCTCTGTCATC
mFabp4	qPCR	AAGGTGAAGAGCATCATAACCCT	TCACGCCTTTCATAACACATTCC
mFoxo1	qPCR	CTACGAGTGGATGGTGAAGAGC	CCAGTTCCTTCATTCTGCACTCG
mLrat	qPCR	ACAAGGAACGCACTCAGAAG	GTCTAGGTGATTGACGAGGATG
mPck1	qPCR	ATCATCTTTGGTGGCCGTAG	CCTCAGATCTCATGGCTGCT
mRarb	qPCR	CTGCTCAATCCATCGAGACAC	CTTGTCCTGGCAAACGAAGC
mRbp1	qPCR	GCGCGCTCGACGTCAAC	ACGATCTCTTTGTCTGGCTTCAG
mRbp4	qPCR	GCAGGAGGAGCTGTGCCTAGA	GGAGGGCCTGCTTTGACAGT
mRetSat	qPCR	CCCATCAAGCAAGGATCCAA	ATGGGTACCAGCGCAGTCA
mRplp0	qPCR	TCATCCAGCAGGTGTTTGACA	GGCACCGAGGCAACAGTT
mTtr	qPCR	CGTACTGGAAGACACTTGGCATT	GAGTCGTTGGCTGTGAAAACC

Appendix Table S3. Antibodies

antibody	product	RRID	application: immunoblotting
ACTB	Santa Cruz #sc-47778 or Cell Sign. #8457	AB_2714189 or AB_10950489	1:1000 in 4% skim milk
ADIPOQ	Cell Sign. #2789	AB_2221630	1:5000 in 4% skim milk
FOXO1	Cell Sign. #2880S (C29H4)	AB_2106495	1:500 in 5% BSA
GAPDH	Cell Sign. #2118 (14C10)	AB_561053	1:2000 in 4% skim milk
HSL	Cell Sign. #4107S	AB_2296900	1:500 in 4% skim milk
RAN	BD Biosciences, #610341	AB_397731	1:2000 in 4% skim milk
RBP4	Agilent/Dako #A0040	AB_2630363	1:500-1:1000 in 4% skim milk
TTR	Dako #A0002		1:333 in 4% skim milk
anti-rabbit 2nd	Thermo Fisher #31460	AB_228341	1:2000 in 4% skim milk
anti-mouse 2nd	Thermo Fisher #31430	AB_228307	1:2000 in 4% skim milk
