# Supplementary figures and images for: Adipocyte HSL is required for maintaining circulating vitamin A and RBP4 levels during fasting
Source: EMBO Rep. 2024 May 20;25(7):8. doi: 10.1038/s44319-024-00158-x (PMC11239848; doi:10.1038/s44319-024-00158-x)

Fig. 1F

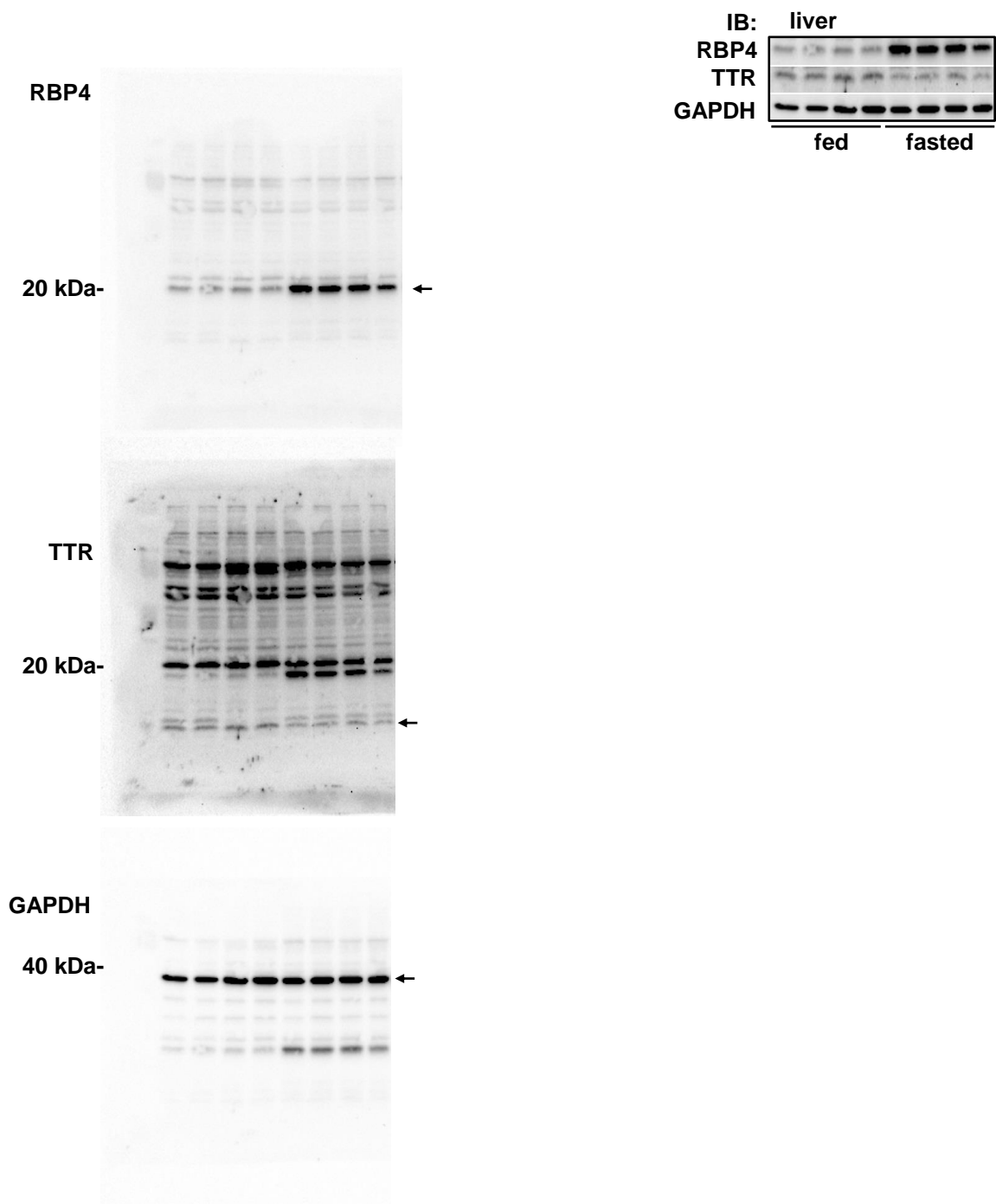

Supplement: Supplementary file 2 — Source data Fig. 1 [file 44319_2024_158_MOESM2_ESM.zip › Figure 1/1F/1F blots.pdf]

Fig. 2A

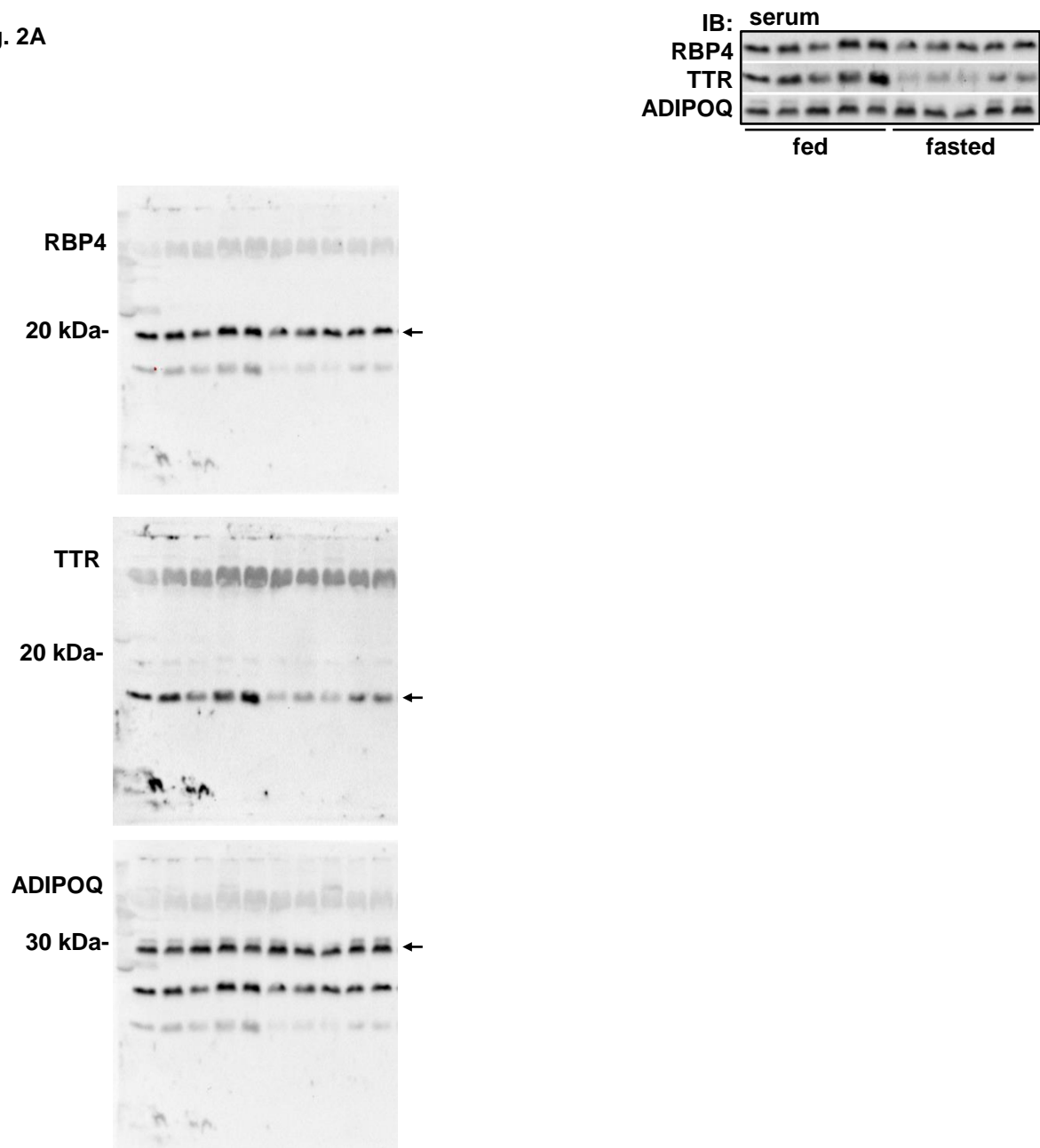

Supplement: Supplementary file 3 — Source data Fig. 2 [file 44319_2024_158_MOESM3_ESM.zip › Figure 2/2A/2A blots.pdf]

Fig. 2B

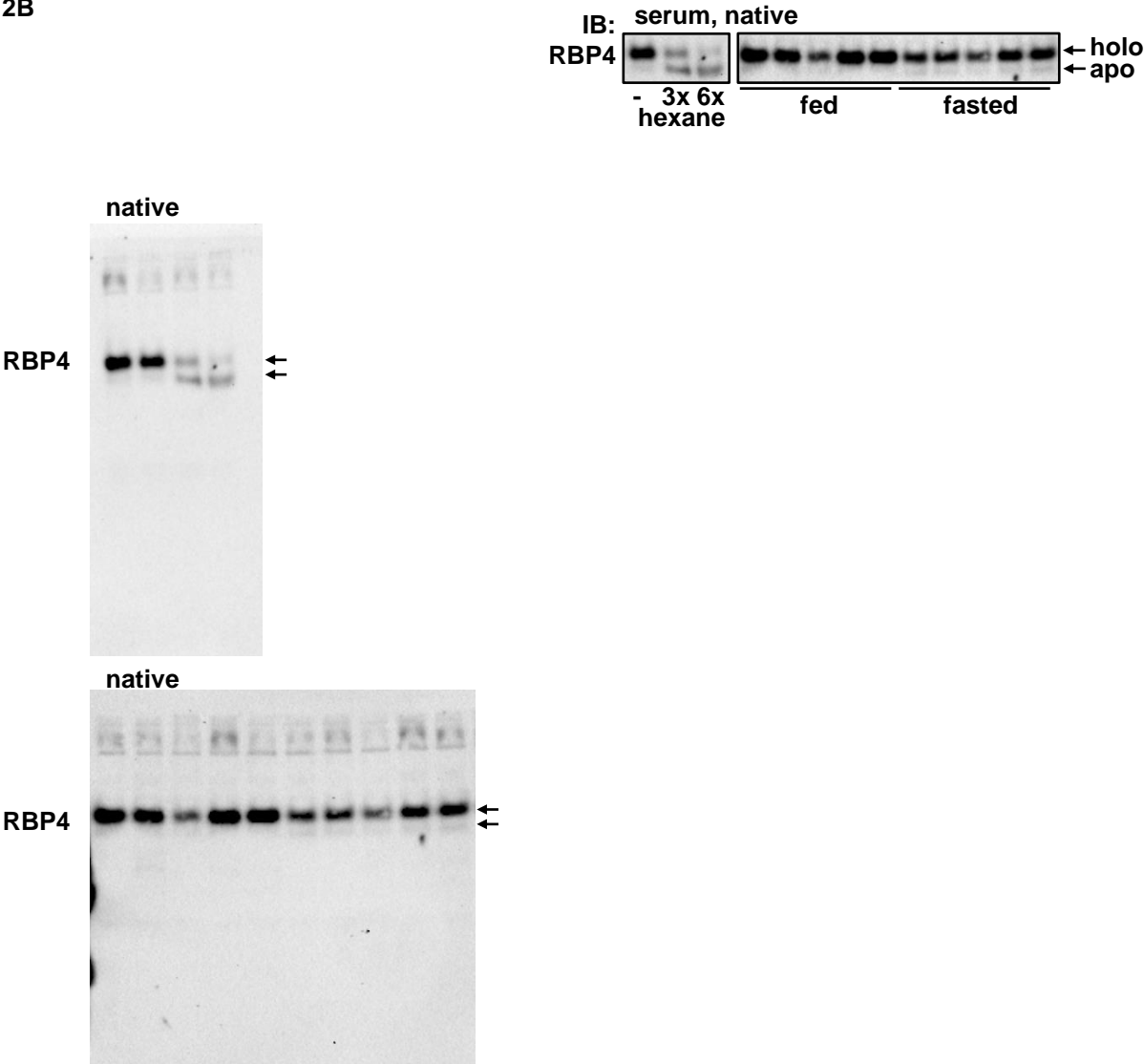

Supplement: Supplementary file 3 — Source data Fig. 2 [file 44319_2024_158_MOESM3_ESM.zip › Figure 2/2B/2B blots.pdf]

Fig. 3E

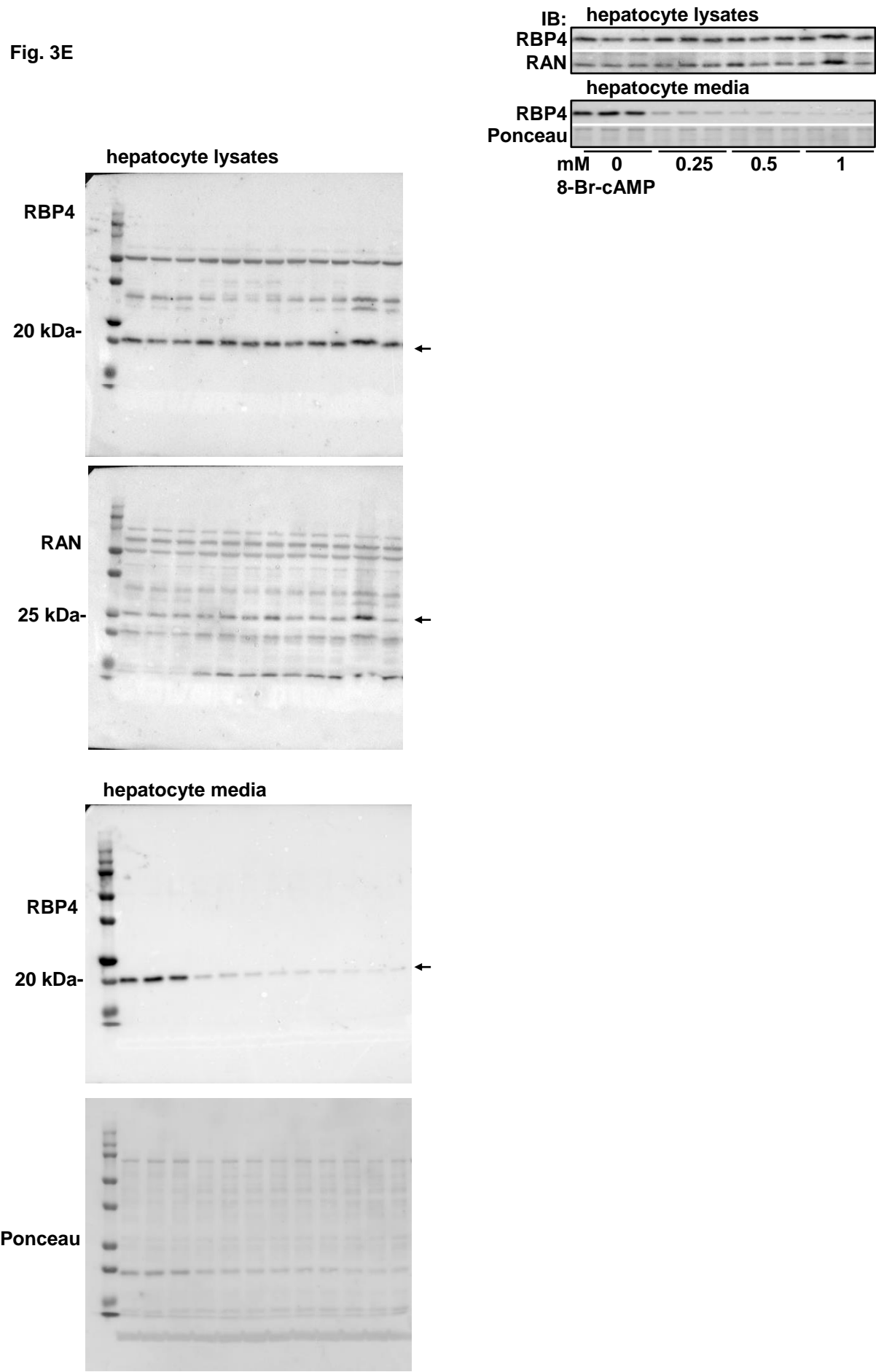

Supplement: Supplementary file 4 — Source data Fig. 3 [file 44319_2024_158_MOESM4_ESM.zip › Figure 3/3E/3E blots.pdf]

Fig. 3G

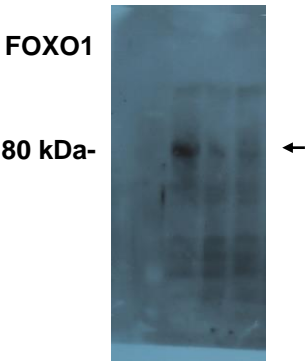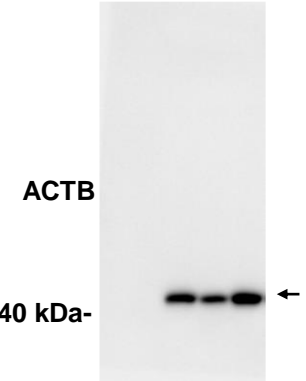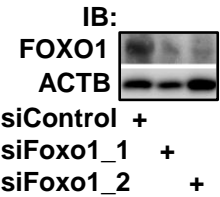

Supplement: Supplementary file 4 — Source data Fig. 3 [file 44319_2024_158_MOESM4_ESM.zip › Figure 3/3G/3G blots.pdf]

Fig. 5E

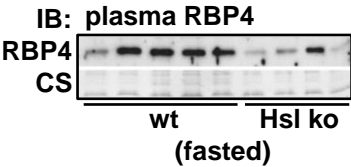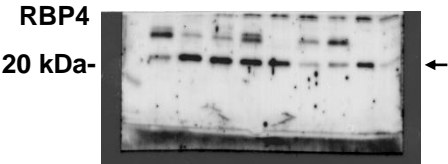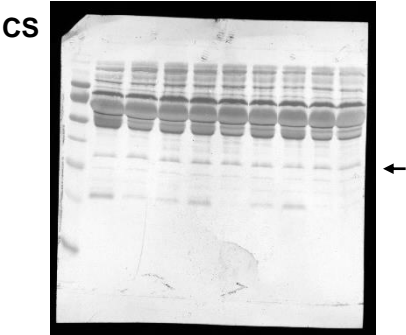

Supplement: Supplementary file 6 — Source data Fig. 5 [file 44319_2024_158_MOESM6_ESM.zip › Figure 5/5E/5E blots.pdf]

Fig. 6A

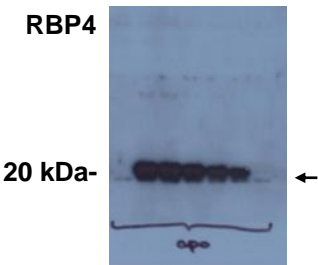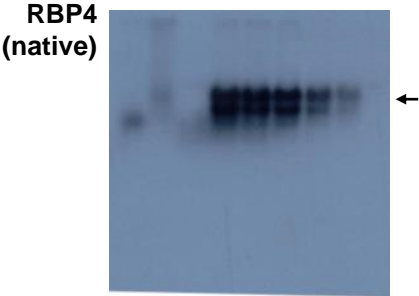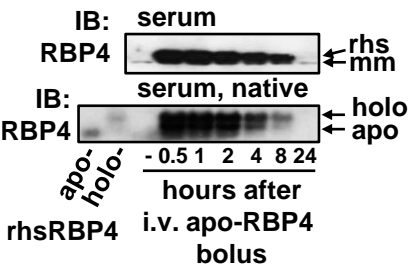

Supplement: Supplementary file 7 — Source data Fig. 6 [file 44319_2024_158_MOESM7_ESM.zip › Figure 6/6A/6A blots.pdf]

Fig. 7C

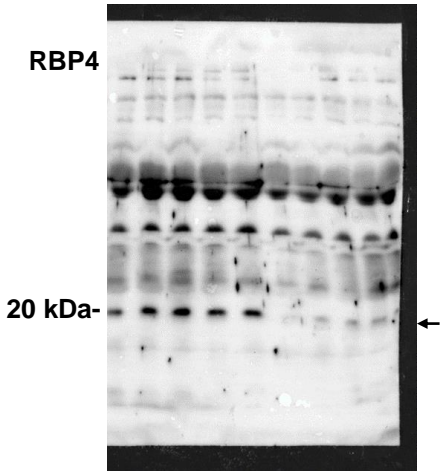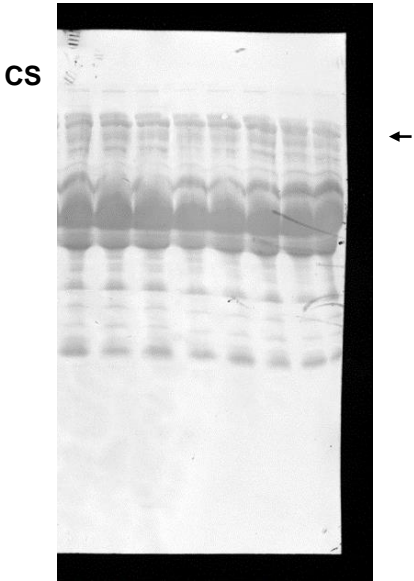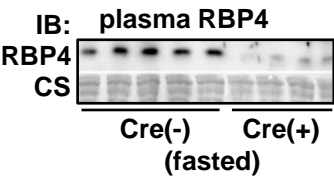

Supplement: Supplementary file 8 — Source data Fig. 7 [file 44319_2024_158_MOESM8_ESM.zip › Figure 7/7C/7C blots.pdf]
