# Supplementary material for: Adipocyte HSL is required for maintaining circulating vitamin A and RBP4 levels during fasting
Source: EMBO Rep. 2024 May 20;25(7):8. doi: 10.1038/s44319-024-00158-x (PMC11239848; doi:10.1038/s44319-024-00158-x)
Supplement: Supplementary file 10 — Expanded View Figures [file 44319_2024_158_MOESM10_ESM.pdf]

Expanded View Figures

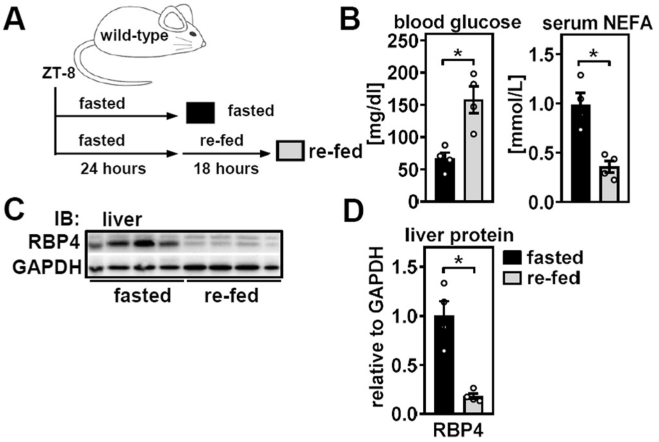

Figure EV1. Refeeding mice reduces RBP4 protein abundance in liver.

(**A**) Mice were fasted or re-fed as depicted. (**B**) Blood glucose and serum NEFA of fasted and re-fed mice were determined. (**C**) Hepatic abundance of RBP4 was determined by immunoblotting, GAPDH served as loading control. (**D**) Densitometric analysis of the blots shown in (**C**). Data information: Data are represented as individual data points of $n = 4$, 4 (**B–D**) biological replicates and mean ± s.e.m. and *$P < 0.05$ vs. fasted mice using an unpaired two-tailed Student's t test.

 © The Author(s)

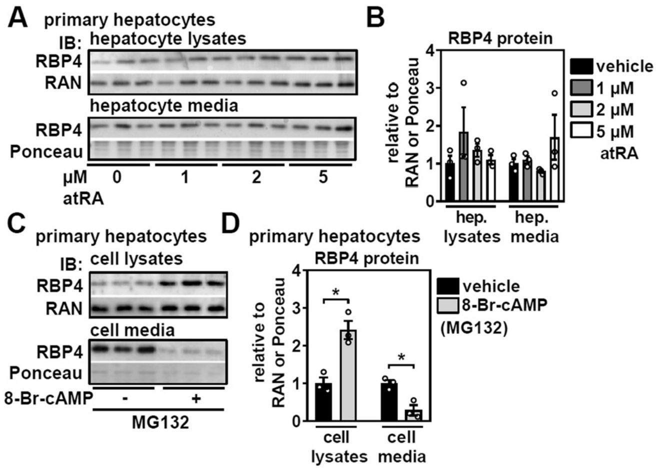

Figure EV2. RBP4 accumulation and secretion in primary hepatocytes is regulated by cAMP signaling but not by all-*trans* retinoic acid (atRA).

(**A**) Primary hepatocytes were incubated with increasing concentrations of atRA and RBP4 protein in cell lysates and media analyzed by immunoblotting, RAN protein and Ponceau membrane staining served as loading controls, respectively. (**B**) Densitometric analysis of blots shown in (**A**). (**C**) Hepatocytes were incubated with 0.5 mM of 8-Br-cAMP for 24 h and RBP4 protein in cell lysates and media analyzed by immunoblotting, RAN protein and Ponceau membrane staining served as loading controls, respectively. 10 μM of the proteasome inhibitor MG132 was added to vehicle and 8-Br-cAMP-treated hepatocytes for the last 4 h before harvesting. (**D**) Densitometric analysis of blots shown in (**C**). Data information: Data are represented as individual data points of $n = 3$ for each condition and mean ± s.e.m. and $^*P < 0.05$ vs. vehicle treatment using an unpaired two-tailed Student's *t* test (**D**).

© The Author(s)

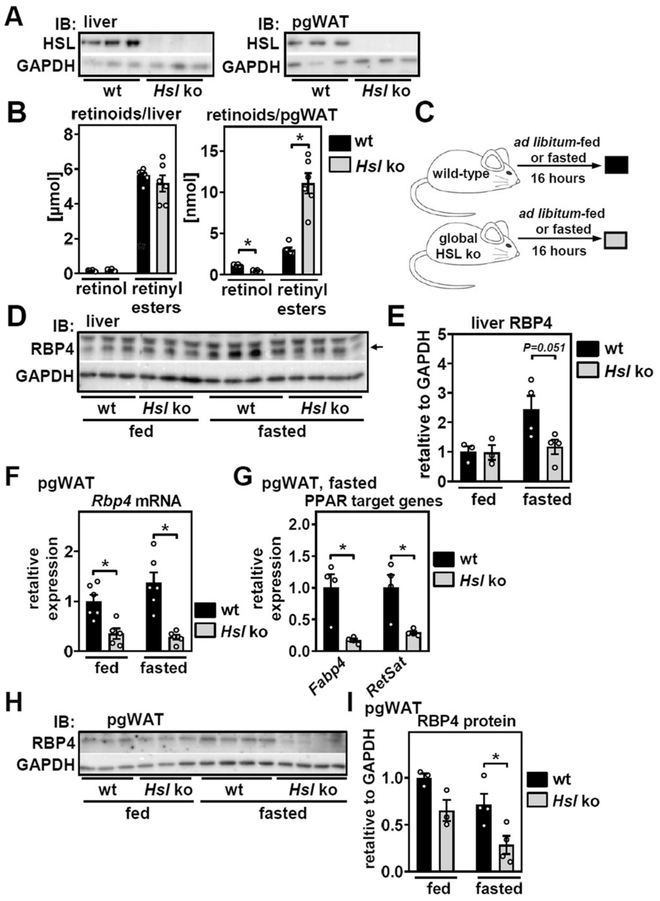

Figure EV3. Global *Hsl* knockout increases retinyl ester content in WAT but not liver and reduces expression of RBP4 in WAT.

(**A**) HSL protein abundance in liver (left panel) and perigonadal white adipose tissue (pgWAT) (right panel) of wild-type (wt) and *Hsl* knockout (ko) mice was determined by immunoblotting. GAPDH protein served as loading control. (**B**) Tissue retinol and retinyl esters in liver and pgWAT were analyzed by HPLC. Retinoids are shown as n/µmol per total organ. (**C**) Wt and *Hsl* ko mice were fed and fasted as depicted and (**D**) abundance of RBP4 protein in livers was determined by immunoblotting. GAPDH served as loading control. (**E**) Densitometric analysis of blots shown in (**D**). (**F**) mRNA expression of *Rbp4* and (**G**) that of canonical PPAR target genes in pgWAT was determined by qPCR. (**H**) Hepatic abundance of RBP4 was determined by immunoblotting, GAPDH served as loading control. (**I**) Densitometric analysis of the blots shown in (**H**). Data information: Data are represented as individual data points of $n = 3, 3$ (**A**), $n = 6, 6$ (**B**), $n = 3, 3, 4, 4$ (**D**, **E**), $n = 6, 5, 6, 6$ (**F**), $n = 4, 4$ (**G**), and $n = 3, 3, 4, 4$ (**H**, **I**) biological replicates and mean ± s.e.m., $^*P < 0.05$ vs. wt mice using an unpaired two-tailed Student's *t* test (**B**, **G**) or a two-way ANOVA with Sidak's correction for multiple testing (**E**, **F**, **I**).

 © The Author(s)

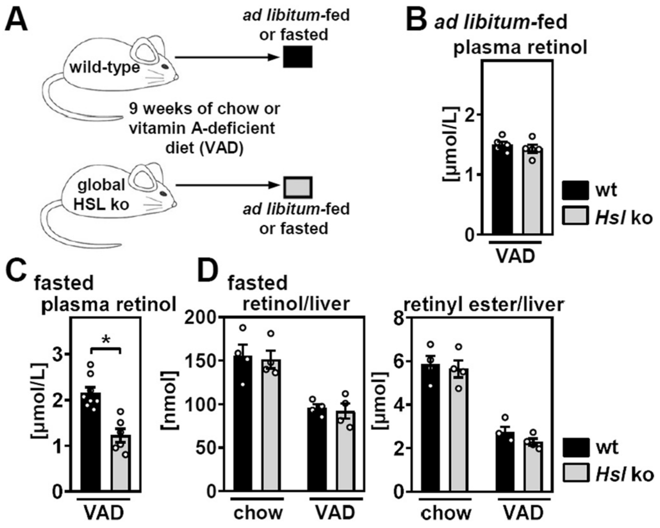

Figure EV4. Global deletion of HSL does not impair hepatic retinol mobilization upon feeding Vitamin A-deficient diet (VAD).

(**A**) Mice of indicated genotype were fed normal chow or VAD and fasted for 16 h or not prior plasma and tissue collection as depicted. (**B**) Plasma retinol in ad libitum-fed mice on VAD for 9 weeks was determined by HPLC. (**C**) Plasma retinol in fasted mice on VAD for 9 weeks was determined by HPLC. (**D**) Retinol (left panel) and retinyl ester content (right panel) of liver after feeding normal chow or VAD in fasted mice was determined by HPLC. Data information: Data are represented as individual data points of $n = 5, 5$ (**B**), $n = 8, 6$ (**C**), and $n = 4$ for each group (**D**) biological replicates and mean ± s.e.m., $^*P < 0.05$ vs. wt mice using an unpaired two-tailed Student's t test (**C**).

© The Author(s)

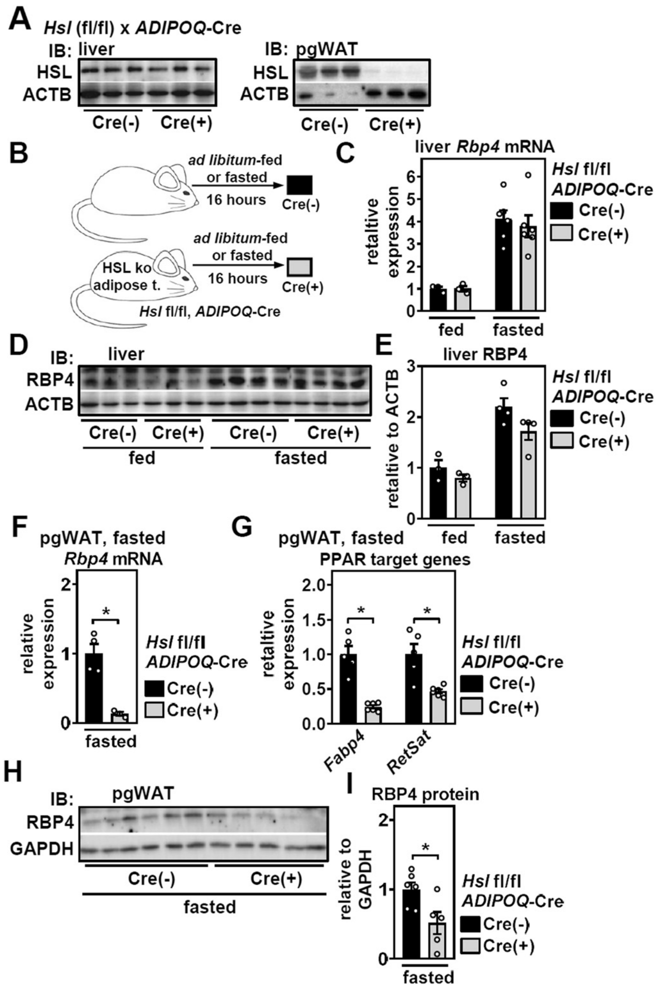

Figure EV5. Adipose tissue-specific *Hsl* knockout does not affect mRNA and protein expression of hepatic RBP4 but reduces RBP4 levels in WAT.

(**A**) HSL protein abundance in liver (left panel) and perigonadal white adipose tissue (pgWAT, right panel) of *ADIPOQ*-Cre(−) and Cre(+) mice with floxed *Hsl* alleles was determined by immunoblotting. ACTB protein served as loading control. (**B**) *ADIPOQ*-Cre(-) and Cre(+) mice with floxed *Hsl* alleles were fasted as depicted and (**C**) hepatic mRNA expression of *Rbp4* determined by qPCR. (**D**) Abundance of RBP4 protein in livers of mice described in (**B**) was determined by immunoblotting. ACTB served as loading control. (**E**) Densitometric analysis of blots shown in (**D**). (**F**) mRNA expression of *Rbp4* and (**G**) that of canonical PPAR target genes in pgWAT of fasted mice was determined by qPCR. (**H**) Abundance of RBP4 in pgWAT of fasted mice was determined by immunoblotting, GAPDH served as loading control. (**I**) Densitometric analysis of the blots shown in (**H**). Data information: Data are represented as individual data points of $n = 3, 3, 6, 6$ (**C**), $n = 3, 3, 4, 4$ (**E**), $n = 4, 3$ (**F**), $n = 5, 6$ (**G**), $n = 6, 5$ (**I**) biological replicates and mean ± s.e.m., *$P < 0.05$ vs. Cre(−) mice using an unpaired two-tailed Student's *t* test (**F**, **G**, **I**).

 © The Author(s)
